# Is the coronary artery calcium score the first-line tool for investigating patients with severe hypercholesterolemia?

**DOI:** 10.1186/s12944-019-1090-8

**Published:** 2019-07-06

**Authors:** Sandra Kutkienė, Žaneta Petrulionienė, Aleksandras Laucevičius, Rimantė Čerkauskienė, Vytautas Kasiulevičius, Artūras Samuilis, Virginija Augaitienė, Aurelija Gedminaitė, Gintarė Bieliauskienė, Akvilė Šaulytė-Mikulskienė, Justina Staigytė, Emilija Petrulionytė, Urtė Gargalskaitė, Eglė Skiauterytė, Gabija Matuzevičienė, Milda Kovaitė, Irena Nedzelskienė

**Affiliations:** 1 Faculty of Medicine Clinic of Cardiac and Vascular Diseases, Vilnius University, Vilnius, Lithuania; 2 Vilnius University Hospital Santaros Klinikos, Children‘s hospital, Vilnius, Lithuania; 3 Faculty of Medicine, Vilnius University, Vilnius, Lithuania; 4 Hospital Santaros Klinikos, Vilnius University, Vilnius, Lithuania; 5 Faculty of Medicine Clinic of Internal Diseases Family Medicine and Oncology, Vilnius University, Vilnius, Vilnius, Lithuania; 6 Department of Radiology Nuclear Medicine and Medical Physics, Vilnius University Institute of Biomechanical Sciences, Vilnius, Lithuania

**Keywords:** Coronary artery calcium score, Cardiovascular risk, Severe hypercholesterolemia, Dyslipidemia, Primary prevention

## Abstract

**Background:**

Coronary artery calcium (CAC) is known as a reliable tool for estimating risk of myocardial infarction, coronary death, all-cause mortality and is even used to evaluate suitable asymptomatic patients. We therefore aimed to evaluate whether CAC scoring can be applied in the algorithm for clinical examination of patients with severe hypercholesterolemia (SH).

**Methods:**

During the period of 2016–2017 a total of 213 asymptomatic adults, underwent computed tomography angiography to evaluate their CAC scoring. The sample consisted of 110 patients with SH and 103 age and sex matched controls without dyslipidemia and established cardiovascular disease.

**Results:**

In total there were 79 (37.2%) subjects with elevated (≥25th) CAC percentiles. Out of them 47 (59.5%) had SH and 32 (40.5%) did not. CAC score did not differ between groups (SH (+) 140.30 ± 185.72 vs SH (−) 87.84 ± 140.65, *p* = 0.146), however there was a comparable difference in how the participants of these groups distributed among different percentile groups (*p* = 0.044). Gender, blood pressure, tabaco use, physical activity, family history of coronary artery disease and diabetes mellitus were not associated with CAC score (*p* > 0.05). There were no significant correlations between biochemical parameters and CAC percentiles except for increase in lipoprotein(a) (*p* = 0.038). Achilles tendon pathology, visceral obesity, body mass index and increased waist-hip ratio were not associated with CAC percentiles either (*p* > 0.05).

**Conclusions:**

CAC score is not associated with presence of SH. CAC score is not an appropriate diagnostic tool in the algorithm for clinical examination of patients with SH. Further larger studies are needed to support our findings.

## Background

In 2016 cardiovascular disease (CVD) remained a major cause of mortality in Lithuania (56.1%) with rates of deaths from coronary heart disease (CHD) being the highest in Europe [1, 2]. Dyslipidemia, hypertension and hyperphosphatemia (in patients with renal disease) all are major clinical risk factors for coronary artery calcification [3]. Estimated prevalence of dyslipidemia in Lithuania is very high [4], it remains an important issue as a slight increase from 89.1 to 89.7% has been observed in a middle-aged population during the period of 2009–2016 [5, 6]. Furthermore, 13.4% (12334) of this population had severe hypercholesterolemia. Dyslipidemia is one of the most important modifiable CVD risk factors known yet [7, 8]. The association between increased lipid concentrations and the risk of CVD is well established [9, 10]. Causal relationship has been strongly supported by epidemiological studies on efficacy of lipid lowering therapy in reducing the incidence of CHD [11].

Even at a young age pathologic evidence of atherosclerosis can be identified soon after risk factor onset, however recognizing the ones at greatest risk and in need of advanced treatment remains a challenge [12]. Coronary artery calcium (CAC) is now established as a reliable tool for estimating risk of myocardial infarction, coronary death and all-cause mortality [13–15]. Guidelines around the world endorse the use of non-contrast cardiac computed tomography (CT) for assessing CAC score among suitable asymptomatic patients in pursue of better clinical risk evaluation [13]. Moreover, cardiac CT for CAC scoring has been validated as an independent prognostic indicator of coronary artery disease (CAD) [16–18]. Although the absence of calcium in the coronary arteries does not rule out atherosclerotic disease it is thought to indicate an excellent long-term prognosis [19, 20].

As prevalence of dyslipidemia is very high in Lithuania, we decided to assess whether CAC scoring can be applied in the algorithm for clinical examination of patients with severe hypercholesterolemia. The aim of our study was to evaluate the benefit of CAC scoring among Lithuanian women and men with severe hypercholesterolemia.

## Methods

During the period of 2016–2017 a total of 213 participants were evaluated. Patients were participants of National Primary Cardiovascular Disease Prevention program. This study included 18–60 year-olds, both men and women. Data was collected with the approval of the Local Research Ethics Committee. Written informed consent was obtained from all participants before their inclusion in the study. Based on to their lipid profile participants were split into severe hypercholesterolemia (110 patients, 51.6%) and control groups (103 patient, 48.4%). Subjects in control group had normal lipid profile and were free of clinically manifested coronary heart disease. To convert CAC score to percentiles we used cut-points as in previous studies [21].

Severe hypercholesterolemia was considered as serum total cholesterol (TC) ≥7.5 mmol/L or low-density lipoprotein-cholesterol (LDL-C) ≥6 mmol/L. If patient had SH and treatment with statins or other drugs was provided but lipid profile was still abnormal (fulfilling criteria of severe hypercholesterolemia), patients were included into the study. We did not differentiated patients into SH without treatment group or SH with insufficient treatment. In Table 1 we present reference ranges for blood lipid parameters that are used in our laboratory. Subjects were not included in severe dyslipidemia group if there were any signs of secondary causes of dyslipidemia (uncontrolled hypothyroidism, diabetes mellitus, nephrotic syndrome, renal insufficiency, cholestasis, viral hepatitis, liver cirrhosis, alcoholism, anorexia), pregnancy, terminal stage cancer and any terminal stage disease. Controlled thyroid dysfunction and diabetes mellitus diagnosed later than the onset of dyslipidemia were not considered as exclusion criteria.

**Table 1:** Reference ranges for blood lipid parameters

Variable	Reference range
Total cholesterol (mmol/L)	< 5.2
Triglycerides (mmol/L)	≤1.8
High-density lipoprotein cholesterol (mmol/L)	> 0.91for men, > 1.2 for women
Low-density lipoprotein-cholesterol (mmol/L)	2,6–3,5
ApoA1 (g/L)	1.1–2.05 for men, 1.25–2.15 for women
ApoB (g/L)	0.55–1.40 for men, 0.55–1.25 for women
ApoA2 (g/L)	0.26–0.51
ApoE (mg/L)	23–63
ApoB/ApoA1	0.35–1.0 for men, 0.30–0.9 for women
Lipoprotein(a) (g/L)	< 0.3

*ApoA1* – apolipoprotein A1, *ApoB* – apolipoprotein B, *ApoA2* – apolipoprotein, *ApoE* – apolipoprotein E

Only subjects without clinically manifested coronary heart disease (myocardial infarction, unstable angina pectoris, stable angina pectoris with positive cardiac stress test, coronary artery pathology identified during cardiac catheterization or coronary computed tomography angiography, coronary artery bypass surgery, percutaneous coronary intervention or acute coronary syndrome), cerebrovascular disease (previous acute ischemic or haemorrhagic stroke, diagnosed stenosis of carotid arteries), peripheral artery disease (acute ischemic syndromes, chronic limb ischemia, aortic aneurysm), dyslipidemia and disorders that may influence blood lipid concentrations (uncontrolled hypothyroidism, diabetes mellitus, nephrotic syndrome, renal insufficiency, cholestasis, viral hepatitis, liver cirrhosis, alcoholism, anorexia), pregnancy, terminal stage cancer and any terminal stage disease were included in control group.

We conducted comprehensive risk profile estimation (history of tobacco use, arterial hypertension, physical activity, dietary habits, body composition analysis). Various diagnostic tests, including coronary artery calcium scoring, echocardiography, abdominal ultrasound, ultrasound of the tendons were performed. Blood cholesterol, apolipoproteins, anthropometric data (height, weight, waist, hip circumference, etc.) as well as heart rate and arterial blood pressure were also evaluated. All the tests and procedures were carried out in the morning and participants were advised not to eat least 12 h before.

Arterial hypertension (AH) was defined as systolic blood pressure ≥ 140 mmHg and/or diastolic blood pressure ≥ 90 mmHg, or the diagnosis of hypertension was documented in a medical record.

Obesity was considered as body mass index (BMI) ≥30 kg/m^2^ and abdominal obesity was determined as waist circumference > 102 cm for men and > 88 cm for women [22]. An increase in waist-hip ratio was considered when it reached > 0.90 for men and > 0.85 for women.

Insufficient physical activity was described as exercises less than 45 min 3 times a week.

Patients were examined in prone position with both feet hanging over the edge of the Table. A linear transducer of 9 MHz was used. The Achilles tendons were accessed from myotendinous junction to the site of the calcaneal insertion in sagittal and transverse planes. Measurements of the tendon thickness (anteroposterior (AP) diameter) were made at the level of the medial malleolus. The Achilles tendons were considered normal if their thickness and echogenicity was uniform in both planes and the AP diameter was less than 6.4 mm for females and 6.8 mm for males. Tendinosis was diagnosed if a fusiform thickening of the Achilles tendon without the disruption of tendon fibers was found with or without intratendinous hypoechoic foci.

Images for CAC scoring were acquired following a standard protocol with 2.5 mm collimation, sequential acquisition and electrocardiographic gating. Imaging was performed with 64 slice multidetector CT (GE LightSpeed VCT, Milwaukee, Wisconsin, US). Advantage Workstation (version 4.6, GE Healthcare, US) software was used for post processing of the images. CAC scores were calculated according to Agatston’s method and later with respect to age, sex, and ethnicity converted to percentiles.

### Statistical analysis

Statistical analysis was performed on IBM SPSS Statistics (version 22.0; SPSS Inc., Chicago, Illinois, USA). Demographic, biochemical, body composition data and data concerning risk factors are presented as mean with standard deviation (± SD) or number (percent) The characteristics between the patient groups were compared using unpaired t-tests or Mann-Whitney tests for continuous variables and Chi-squared tests for categorical data. A 2-tailed *p*-value of < 0.05 was considered significant.

## Results

**Fig. 1:**
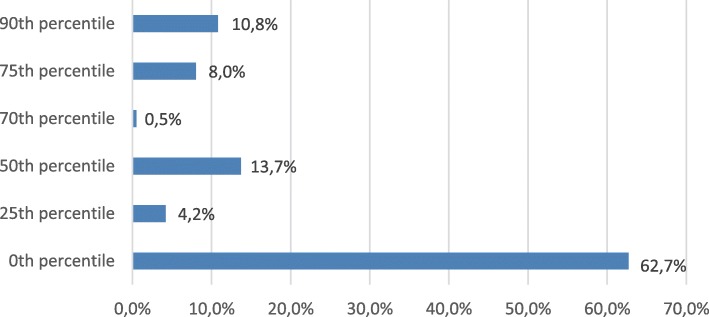
Distributions of all participants according to CAC score percentiles

The average age of subjects was 49.15 ± 8.01 years. The sample consisted of 105 (49.3%) women and 108 (50.7%) men. Fig. 1 demonstrates distribution of participants according to their CAC score percentiles. Table 2 represents baseline lipid profile and apolipoproteins of subjects with CAC score ≥ 25th percentile. There were no significant correlations between biochemical parameters and CAC percentiles except for lipoprotein(a). Increase in lipoprotein(a) was associated with CAC score percentiles (*p* = 0.038) (Table 2).

**Table 2:** Associations between CAC percentiles and lipid profile components

Variable	25th percentile	50th percentile	75th pecentile	90th percentile	*P* value	Chi square
ApoA1 F < 1,25 g/L; M < 1,1 g/L	0%	6.9%	11.8%	13.0%	0.775	1.787
ApoA2 < 0,26 g/L	33.3%	6.9%	0%	13.0%	0.095	7.901
ApoB/ApoA1 F > 0.9; M > 1.0	25.0%	34.5%	47.1%	52.2	0.479	3.495
ApoB F > 1.25 g/L; M > 1.4 g/L	33.3%	41.4%	64.7%	52.2%	0.371	4.268
ApoE > 63 mg/L	25.0%	46.4%	41.2%	59.1%	0.413	3.950
TC > 5 mmol/L	66.7%	86.2%	82.4%	78.3%	0.219	5.743
Lipoprotein(a) > 0.3 g/L	11.1%	31.0%	0%	39.1%	0.038	10.163
LDL-C > 3 mmol/L	55.6%	75.9%	70.6%	69.6%	0.772	1.804
HDL-C reduction	33.3%	37.9%	17.6%	21.7%	0.258	5.294
TG F < 1,2 mmol/L; M < 1,0	44.4%	51.7%	41.2%	52.2%	0.808	1.607

**Fig. 2:**
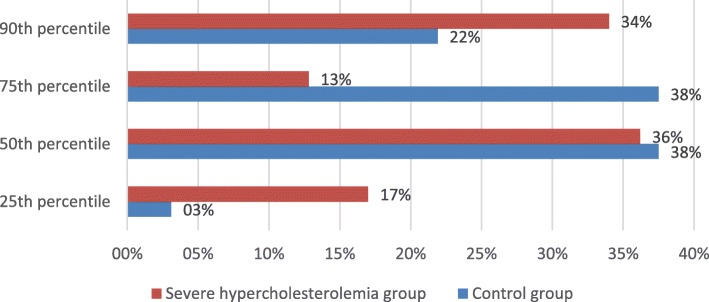
Distribution of CAC score ≥25th percentile in severe hypercholesterolemia and control groups

**Fig. 3:**
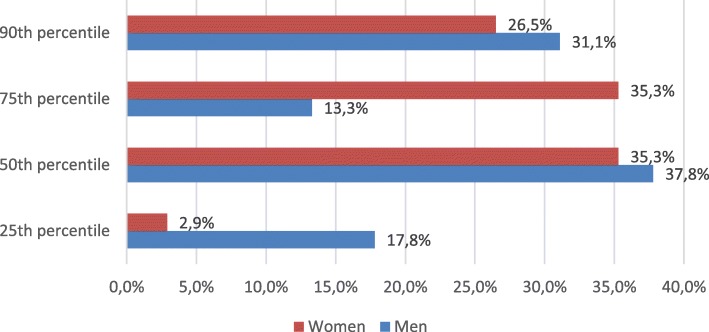
Distribution of CAC score ≥25th percentile according to gender

In 0th CAC percentile group 62 (47%) subjects out of 133 had severe hypercholesterolemia. In total there were 79 (37.2%) subjects with elevated (≥25th) CAC percentiles. Out of them 47 (59.5%) had severe hypercholesterolemia and 32 (40.5%) did not. Even though CAC score did not differ between groups (severe hypercholesterolemia (+) 140.30 ± 185.72 vs severe hypercholesterolemia (−) 87.84 ± 140.65, *p* = 0.146) (Table 3), there was a comparable difference in how the participants of these groups distributed among different percentile groups (*p* = 0.044) according to their age, gender, race/ethnicity (Fig. 2). However, gender was not associated with change in distribution of CAC percentiles (*p* = 0.075) (Fig. 3).

**Table 3:** Distribution of CAC score ≥ 25th percentile between severe hypercholesterolemia and control groups

Variable – CAC score	Severe hypercholesterolemia	Control group	p value
All subjects (*n* = 213)	140.30 ± 185.72	87.84 ± 140.65	0.146
Women (*n* = 105)	157.67 ± 215.25	49.50 ± 60.75	0.131
Men (*n* = 108)	134.34 ± 177.58	96.69 ± 152.83	0.429

CAC score – coronary artery calcium score

**Fig. 4:**
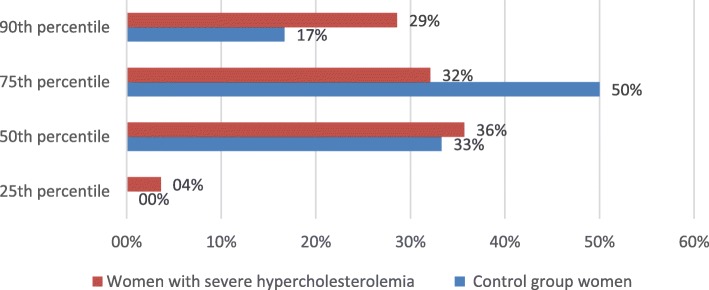
Distribution CAC score ≥25th percentile between severe hypercholesterolemia and control groups in women

**Fig. 5:**
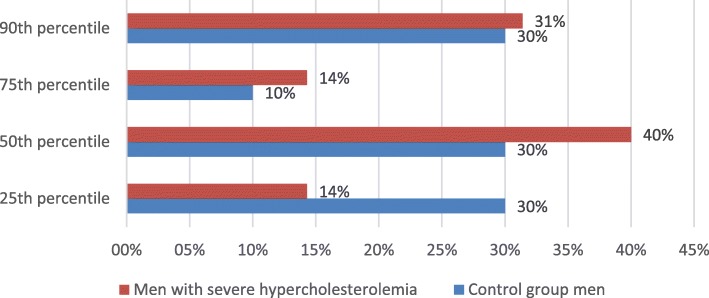
Distribution of CAC score ≥25th percentile between severe hypercholesterolemia and control groups in men

**Fig. 6:**
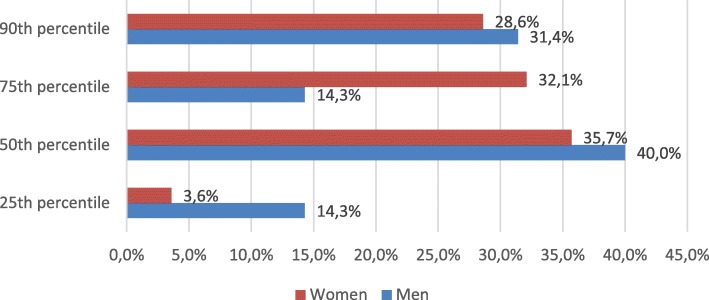
Distribution of CAC score ≥25th percentile in severe hypercholesterolemia group between men and women

Figures 4 and 5 represent distribution of CAC percentiles by gender. Neither women nor men demonstrated percentile differences between severe hypercholesterolemia and control groups (women *p* = 0.272, men *p* = 0.706). There were no gender differences in severe hypercholesterolemia group separately as well (*p* = 0.238) (Fig. 6).

CAC score did not differ between hypertensive and normotensive patients (122.13 ± 171.57 vs 116.05 ± 170.44 respectively, *p* = 0.875), smokers and non-smokers (178.00 ± 202.54 vs 100.38 ± 155.52 respectively, *p* = 0.083), subjects with and without family history of coronary artery disease (110.61 ± 159.50 vs 132.83 ± 187.72 respectively, *p* = 0.576). CAC score was not associated with physical activity (PA) (insufficient PA group 130.04 ± 179.09 vs sufficient PA group 104.50 ± 158.44, *p* = 0.512) or family history of diabetes mellitus (group with family history of diabetes 197.40 ± 285.85 vs group without family history of diabetes 100.96 ± 125.65, *p* = 0.219) as well.

**Fig. 7:**
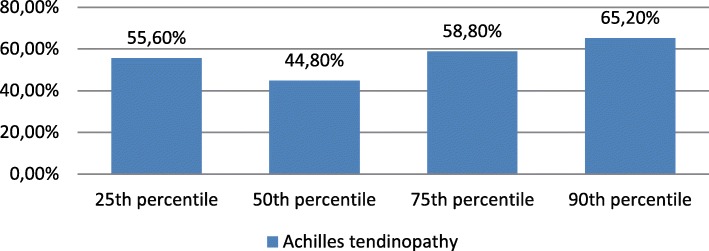
Distribution of Achilles tendinopathy between CAC score ≥25th percentile

Associations between Achilles tendon pathology and CAC score percentiles are represented in Fig. 7. CAC percentiles did not differ between subjects with and without ultrasonographically evident Achilles tendinopathy (*p* = 0.480).

**Fig. 8:**
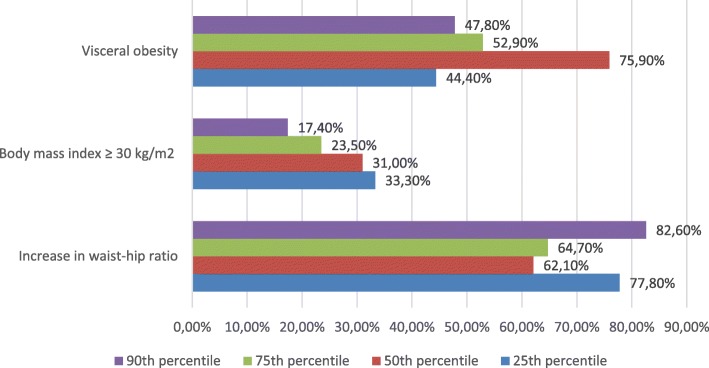
Association between body composition analysis and elevated CAC score ≥25th percentile

Furthermore, body composition analysis did not reveal any significant association between CAC percentiles and visceral obesity (*p* = 0.17), body mass index (*p* = 0.20) or increased waist-hip ratio (*p* = 0.25) as well (Fig. 8).

## Discussion

Although CAC score is an independent predictor of coronary events [22–24] and is useful in acute coronary events risk classification [24, 25], it is not yet known whether screening asymptomatic patients for coronary calcification should be recommended [13, 26] due to the high prevalence of calcification in asymptomatic patients [27]. In this study, we investigated a large group of subjects with severe hypercholesterolemia and genetic defects may be responsible for the severity of it. Familial hypercholesterolemia is characterized by extremely elevated LDL-C levels that lead to atherosclerotic plaque deposition in the coronary arteries and proximal aorta at an early age [28]. Our study showed that CAC score was comparable between control and severe hypercholesterolemia groups and thus is not associated with severe hypercholesterolemia. Nevertheless, when converted into percentiles it demonstrated a significant association. As our study was cross-sectional and included relatively low number of patients, there was a possibility to get different results than in ACC/AHA new lipid guidelines. Our conclusions were based on our data and population nevertheless CAC predictable values are verified in guidelines.

According to our results, CAC score is not the best first line tool in the algorithm for clinical examination of patients with severe hypercholesterolemia. As SH might exist in patients who are relatively young and according to Shaw LJ et al. there is a direct relationship between coronary artery calcium and age [29] our patients might have been too young for coronary artery calcification since our study included only 18–60-year-olds. What is more, there is a number of patients with familial hypercholesterolemia in our SH group but only few of them could be homozygous. As a second line tool for SH patients CAC score measurement is reasonable to perform in order to predict progression of disease, to evaluate effectiveness of treatment and patient’s prognosis.

Even though coronary artery calcification is a marker of coronary atheroma [30], the relationship with acute coronary events is yet not clear. The absence of vascular calcification does not exclude the presence of coronary artery disease [19]. Macrocalcifications seen on a clinical CT evolve from microcalcifications [26]. Microscopic calcifications can be caught at histopathology [31, 32] but are too small to be visible on clinical CT with a spatial resolution of approximately 0.5 mm [33]. Multiple microcalcifications may appear on a CT scanning as “spotty” calcifications. Microcalcifications and spotty calcifications may be associated with plaque rupture [31] or progression of atheroma volume [34], whereas macroscopic calcification is more likely to be associated with lesion stabilisation [26, 35–41].

Worldwide clinical practice guidelines for dyslipidemia emphasize allocating statin therapy to those at the highest absolute atherosclerotic CVD risk [42]. Studies that are examining the relationship between statins and calcification of the coronary arteries are ambiguous, with results varying from increase to decrease, or no change in coronary calcification at all [26]. Statins’ possible procalcific effect on atheroma is consistent with its possible plaque-stabilizing effect [26, 43, 44]. It has even been suggested that CAC score could be used to guide statin therapy if the use of these drugs has a significant effect on quality of life or is costly [45].

We found no significant difference in how CAC percentiles distributed between men and women neither in general group nor in group with severe hypercholesterolemia only. However gender difference has been reported in previous studies, with data suggesting that men are more prone to greater calcification of the coronary arteries compared to women [46].

Lipoprotein(a) and coronary artery calcification are both considered to be associated with cardiovascular disease [22–24, 47, 48]. Lipoprotein(a) was shown to positively correlate with CAC score [49] and higher values were discovered to indicate CAC score progression over a four year period [50]. Our results were consistent with previous studies and showed correlation between levels of lipoprotein(a) and different CAC percentiles.

J. Park et al. has demonstrated that body mass index is positively associated with CAC score [51]. S. Y Jang et al. has also shown that body mass index, waist circumference and waist-hip ratio are significantly related to CAC score [52]. However, we did not find body mass index and waist-hip ratio to correlate with CAC score percentiles.

Achilles tendon xanthomas (TX) have been associated with greater risk of premature cardiovascular disease [37, 53, 54]. TX have also been considered to be a hallmark and a diagnostic criteria for familial hypercholesterolemia [39, 54, 55]. Detection of TX is likely to indicate a possible genetic defect in lipid metabolism and therefore a higher risk for CVD [37, 54, 56–59]. In a study performed by L. C. Mangili et al. Achilles TX have been independently associated with extension of subclinical coronary atherosclerosis quantified by computed tomography CAC score in familial hypercholesterolemia patients [60]. However, we did not find Achilles tendon pathology to be associated with CAC percentiles.

Our study did not find traditional CVD risk factors to correlate with distribution of CAC score percentiles. However, there are studies suggesting that LDL-C and systolic blood pressure are independent predictors of adulthood CAC [61] and CAC scoring could be considered in symptomatic young men with diabetes mellitus [62].

Nonetheless, our results must be interpreted with caution because there may be some possible limitations in this study. The primary limitation to the generalization of these results is relatively moderate number of study population. It led us to restriction of dividing population into subgroups and analyzing smaller variables and tendencies in between. Groups of the study were found to be quite heterogeneous especially according to wide range of age. Time interval among patients could determine differences of theirs comorbidities which have impact on CAC score. What is more, different laboratories and investigators of the patients could also have minor influence to the study results. Further researches could help follow up patients’ CAC score and overall health to evaluate CAC score dynamics and relations to other factors, particularly SD.

## Conclusions

CAC score is not associated with presence of severe hypercholesterolemia. CAC score is not an appropriate diagnostic tool in the algorithm of severe hypercholesterolemia examination. Further larger studies are needed to support our findings.

## Data Availability

The datasets used and analysed during this study are available from the corresponding author on reasonable request.

## Abbreviations

AH: Arterial hypertension
AP: Anteroposterior
ApoA1: Apolipoprotein A1
ApoA2: Apolipoprotein
ApoB: Apolipoprotein B
ApoE: Apolipoprotein E
BMI: Body mass index
CAC: Coronary artery calcium score
CAD: Coronary artery disease
CHD: Coronary heart disease
CT: Computed tomography
CVD: Cardiovascular disease
LDL-C: Low-density lipoprotein-cholesterol
SD: Standard deviation
TC: Serum total cholesterol
TG: Triglycerides
TX: Tendon xanthomas
